# Facilitators and barriers to the implementation of new critical care practices during COVID-19: a multicenter qualitative study using the Consolidated Framework for Implementation Research (CFIR)

**DOI:** 10.1186/s12913-023-09209-w

**Published:** 2023-03-20

**Authors:** Santana Silver, Kayla Christine Jones, Sarah Redmond, Emily George, Sarah Zornes, Amelia Barwise, Aaron Leppin, Yue Dong, Lori A. Harmon, Vishakha K. Kumar, Christina Kordik, Allan J. Walkey, Mari-Lynn Drainoni

**Affiliations:** 1 Evans Center for Implementation & Improvement Sciences (CIIS), Department of Medicine, Boston University School of Medicine, 72 East Concord St, Boston, MA 02118 USA; 2 Robert D. and Patricia E. Kern Center for the Science of Health Care Delivery, Mayo Clinic Rochester, 200 First Street SW, Rochester, MN 55905 USA; 3 Boston University School of Public Health, 715 Albany St, Boston, MA 02118 USA; 4 Division of Pulmonary and Critical Care Medicine, Mayo Clinic, 200 First Street SW, Rochester, MN 55905 USA; 5 Mayo Center for Clinical and Translational Science (CCaTS), 200 First Street SW, Rochester, MN 55905 USA; 6 Department of Anesthesiology and Perioperative Medicine, Mayo Clinic, 200 First Street SW, Rochester, MN 55905 USA; 7 Department of Research and Quality, Society of Critical Care Medicine, 500 Midway Drive, Mount Prospect, IL 60056 USA; 8 The Pulmonary Center, Division of Pulmonary, Allergy, Critical Care and Sleep Medicine, 72 E. Concord St Housman (R), Boston, MA 02118 USA; 9 Section of Infectious Diseases, Boston University School of Medicine, 801 Massachusetts Avenue, Room 2014, Boston, MA 02118 USA; 10 Department of Health Law, Policy & Management, Boston University School of Public Health, 801 Massachusetts Avenue, Room 2014, Boston, MA 02118 USA

**Keywords:** COVID-19, Critical care, Implementation science, CFIR, Barriers, Facilitators

## Abstract

**Background:**

The COVID-19 pandemic produced unprecedented demands and rapidly changing evidence and practices within critical care settings. The purpose of this study was to identify factors and strategies that hindered and facilitated effective implementation of new critical care practices and policies in response to the pandemic.

**Methods:**

We used a cross-sectional, qualitative study design to conduct semi-structured in-depth interviews with critical care leaders across the United States. The interviews were audio-taped and professionally transcribed verbatim. Guided by the Consolidated Framework for Implementation Research (CFIR), three qualitative researchers used rapid analysis methods to develop relevant codes and identify salient themes.

**Results:**

Among the 17 hospitals that agreed to participate in this study, 31 clinical leaders were interviewed. The CFIR-driven rapid analysis of the interview transcripts generated 12 major themes, which included six implementation facilitators (i.e., factors that promoted the implementation of new critical care practices) and six implementation barriers (i.e., factors that hindered the implementation of new critical care practices). These themes spanned the five CFIR domains (Intervention Characteristics, Outer Setting, Inner Setting, Characteristics of Individuals, and Process) and 11 distinct CFIR constructs. Salient facilitators to implementation efforts included staff resilience, commitment, and innovation, which were supported through collaborative feedback and decision-making mechanisms between leadership and frontline staff. Major identified barriers included lack of access to reliable and transferable information, available resources, uncollaborative leadership and communication styles.

**Conclusions:**

Through applying the CFIR to organize and synthesize our qualitative data, this study revealed important insights into implementation determinants that influenced the uptake of new critical care practices during COVID-19. As the pandemic continues to burden critical care units, clinical leaders should consider emulating the effective change management strategies identified. The cultivation of streamlined, engaging, and collaborative leadership and communication mechanisms not only supported implementation of new care practices across sites, but it also helped reduce salient implementation barriers, particularly resource and staffing shortages. Future critical care implementation studies should seek to capitalize on identified facilitators and reduce barriers.

**Supplementary Information:**

The online version contains supplementary material available at 10.1186/s12913-023-09209-w.

## Background

The coronavirus disease 2019 (COVID-19) pandemic has impacted social, political, and economic structures and strained healthcare systems across the world. Critical care units have been especially overburdened, tasked with identifying best critical care practices amidst unprecedented uncertainty, fear, a rapidly changing evidence base, and strained resources [1].

As the quality of critical care is instrumental in reducing and preventing complications and deaths due to the pandemic, it is crucial to identify and implement best care practices to promote optimal outcomes for high acuity COVID-19 patients. Implementation science, which seeks to rigorously develop and evaluate implementation strategies to adopt and integrate best practices, is uniquely situated to assess, improve, and sustain implementation strategies for effective uptake of new care practices in stressed intensive care units (ICUs) during domestic and international health emergencies.

Strategies used by clinical leaders to adapt to new care guidelines and implement practice changes in the early stages of COVID-19 are unclear. For example, methods for engaging hospital leaders with practices changes and providing frontline staff with material and emotional support to meet the demands of frequent practice changes are unknown. To this end, the primary purpose of this study was to qualitatively explore facilitators and barriers to the implementation of new critical care practices necessitated by COVID-19 that were consistent across hospitals with varied COVID-19 mortality rates. A follow-up study aims to investigate whether certain implementation factors were associated with variability in mortality. Improving our understanding of changes to typical ICU clinical practices when caring for COVID-19 patients, how and why these changes were made, and facilitators and barriers to the integration of these changes will provide valuable insight into how to best support clinical leadership as they adopt new life-saving practices.

## Methods

### Study design and conceptual framework

We used a cross-sectional, qualitative study design to investigate ICU responses to COVID-19. Qualitative data were collected through semi-structured, in-depth interviews with ICU clinical leaders (medical and nursing directors) and analyzed using rapid analytic methods. Damschroder et al.’s Consolidated Framework for Implementation Research (CFIR) was used to guide data coding and analysis [2]. The CFIR is a meta-theorical framework used by implementation scientists to systematically identify factors that may emerge in various, multi-level contexts that influence implementation [3–5]. CFIR incorporates 34 constructs across five domains, including ‘Intervention Characteristics’, ‘Inner Setting’, ‘Outer Setting’, ‘Characteristics of Individuals’, and ‘Process’. Evidence has shown that the constructs included in each domain are key factors that affect implementation effectiveness [2]. Using the CFIR provided a systematic way to identify key influences on implementation of new practices, organize the qualitative data, and group descriptions of implementation lessons learned into distinct categories.

### Facility selection

Participating facilities were identified using an international COVID-19 registry – Society of Critical Care Medicine’s (SCCM) Discovery Viral Infection and Respiratory Illness Universal Study COVID-19 Registry (Discovery VIRUS COVID-19 Registry) – that tracks current critical care patterns and determine the variations in practice across U.S. hospitals [6, 7]. Variation in mortality from respiratory failure due to COVID-19 has previously been described [8]. Hospital risk-adjusted mortality rates for patients receiving mechanical ventilation for COVID-19 were calculated from analysis of 4749 patients within 84 hospitals enrolling more than 10 patients in the Registry with complete outcome data between February 15, 2020, and November 30, 2020 [8]. For the purposes of this study, hospitals and the study team were blinded to risk adjusted mortality quartile, which was used as a measure of hospital performance during early COVID-19. Twenty hospitals with the 10 highest and 10 lowest adjusted mortality quartiles were invited to participate in order to address the overarching research aim to identify implementation practices and factors that were both consistent (this study) and different (subsequent study) across high and low performing hospitals.

### Study participant recruitment procedure

ICU clinical leaders were recruited from the participating hospitals using a purposive sampling technique to ensure we had participants from hospitals within both the highest and lowest adjusted mortality quartiles. During recruitment, we emailed all ICU medical and nursing directors who were identified by local facility principal investigators of the Discovery VIRUS COVID-19 Registry. A researcher (EG) conducted video conference calls with candidates who responded via email to explain the research purpose and confirm eligibility. To be eligible, candidates had to be English-speaking and in a current ICU clinical leadership role since January 2019. This ensured that interviewees had both pre- and post-COVID-19 experience, which was necessary to investigating how new critical care guidelines were implemented in response to the pandemic. If eligible, participants were informed of the voluntary nature of study participation and were verbally consented at the end of the eligibility screening video conference call. The Boston Medical Center/Boston University Medical Center Institutional Review Board (IRB) approved all study procedures.

### Data collection

A semi-structured interview guide was designed by the study team and is provided in Additional file 1. We asked 13 open-ended questions with probes to elicit rich descriptions from participants related to ICU practice changes and implementation factors that influenced the uptake of changes. For example: *How did COVID-19 affect your usual ICU practices? Which practice changes were easiest/hardest to implement and what made these changes easy/difficult to implement? In thinking about all the changes you’ve had to make, what strategies, tools and/or resources have been the most helpful?* One trained qualitative researcher (EG) conducted 31 individual semi-structured qualitative interviews between February and May 2021. Interviews lasted 45-60 minutes and were conducted over the Zoom video-conferencing platform, audio-recorded, and professionally transcribed verbatim. Upon completion of their interviews, participants received an honorarium gift card of $50 for their time and efforts.

### Data analysis

A consensual qualitative analytic approach was used to guide the directed content analysis [9, 10]. Given our study aim was to provide timely and actionable information to ICU clinical leadership, we used rapid evaluation methods that have been previously described in the literature [11–15]. Specifically, we conducted a template analysis of interview transcripts, a type of thematic analysis that has a relatively high degree of structure and speed through utilization of a coding template that is based on a priori themes [16]. To derive a priori themes, we applied the CFIR as the primary coding framework, allowing us to organize the qualitative data into salient constructs that studies have identified as key influences of implementation effectiveness [2]. The publicly available CFIR codebook template was used to develop an initial coding template specific to this study. To identify relevant CFIR domains and constructs to include in the initial codebook, the qualitative lead (SS) mapped each interview question to applicable factors across the framework (Table 1).

**Table 1:** Example interview guide questions and related CFIR domains/constructs

Interview Guide Question	CFIR Domain(s)	CFIR Construct(s)
*How did covid affect your usual ICU practices?*	Inner Setting	Tension for Change [Renamed: Necessity of Change]
*When changing these specific practices, how were these decisions made? Who was involved in making these decisions and why?*	Process	Formally appointed internal implementation leaders
*In thinking about all the changes you’ve had to make, what strategies, tools and/or resources have been the most helpful?*	Inner Setting	Networks & Communications; Leadership Engagement; Available Resources
*Which practice changes were easiest/hardest to implement? What made these changes easy/difficult to implement?*	Intervention characteristics; Inner Setting	Complexity; Available Resources
*What could your institution do to be more responsive to the needs of covid patients?*	Outer setting	Needs & Resources of Those Served by the Organization [Renamed: Needs of Patients]
*How did institutional regulations or external guidelines effect the way you provided care to covid patients?*	Intervention characteristics	Innovation Source
*How were these changes communicated to staff?*	Inner Setting; Process	Networks & Communications; Access to Knowledge & Information; Key Stakeholders [Renamed: Engaging Staff]
*How did the staff respond to these changes?*	Inner Setting; Characteristics of Individuals	Implementation Climate; Knowledge & beliefs about the innovation [Renamed: Knowledge & beliefs about changes]

*CFIR* Consolidated Framework for Implementation Research

**Fig. 1:**
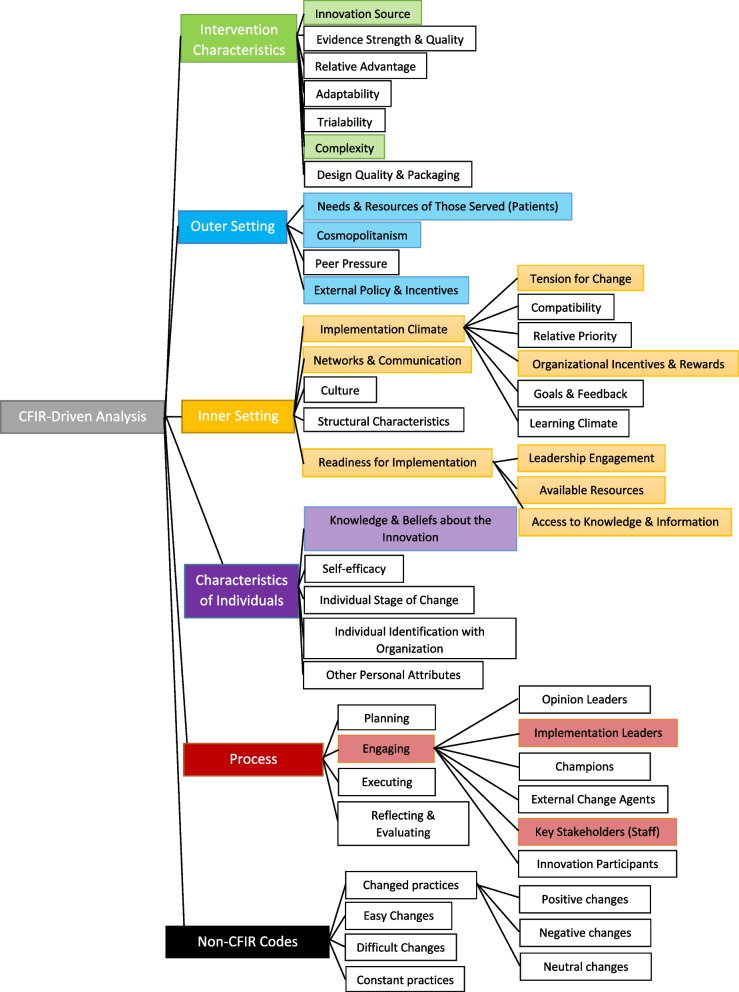
Final Coding Tree: based on CFIR domains/constructs and non-CFIR codes

**Fig. 2:**
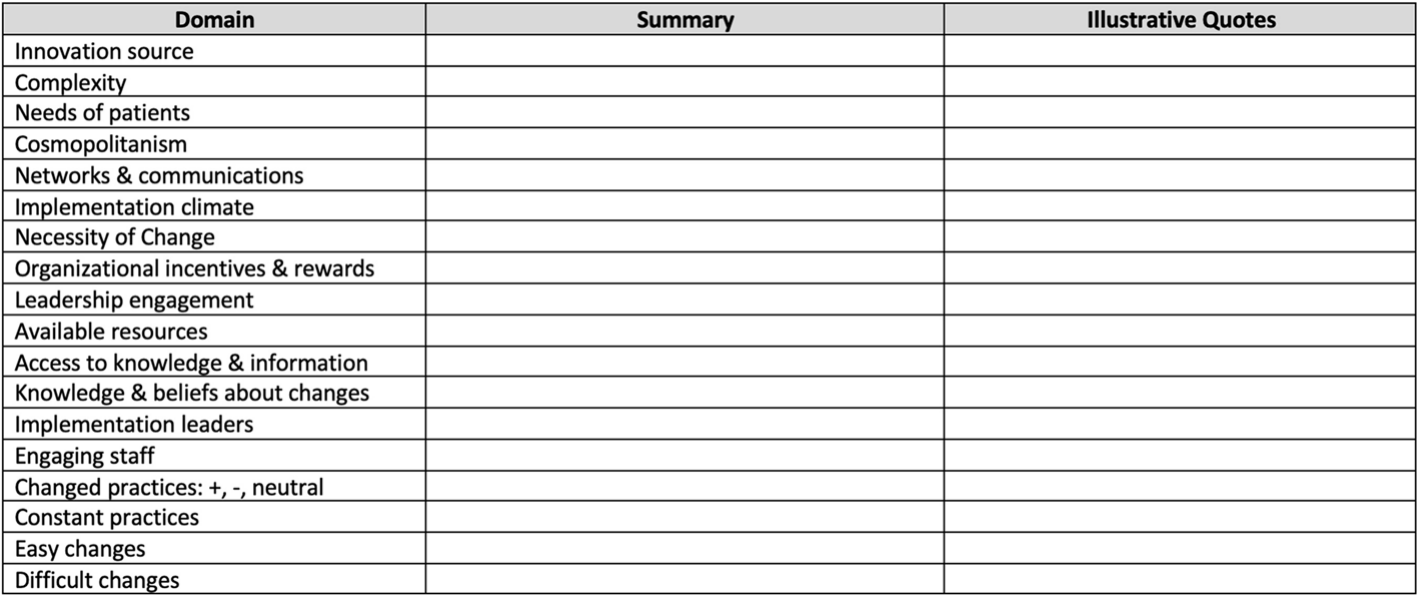
Templated Summary Table: used to summarize each interview transcript

The evaluation team, consisting of three data coders (SS, SR, KCJ), tested the codebook with two interview transcripts. It was modified based on feedback, including the addition of non-CFIR codes that were identified through reviewing the subset of data (See Fig. 1 for the final coding tree). A templated summary table was constructed by the qualitative lead using the pre-specified CFIR codes and inductively generated non-CFIR codes (Fig. 2).

Using the finalized codebook and templated summary table, the evaluation team followed a consensus approach to develop a memo report for each facility. First, the three coders independently coded a single transcript and populated the templated summary table in Microsoft Word with data extracted from interview transcripts, including summaries of key points and illustrative quotes for each CFIR construct. The researchers then met to compare their summary tables, discuss differences, and agree on final consensus summaries. This process was repeated with three additional transcripts, after which the analysts had reached high inter-coder reliability. The remaining 27 interview transcripts were divided for single-coding analysis.

**Fig. 3:**
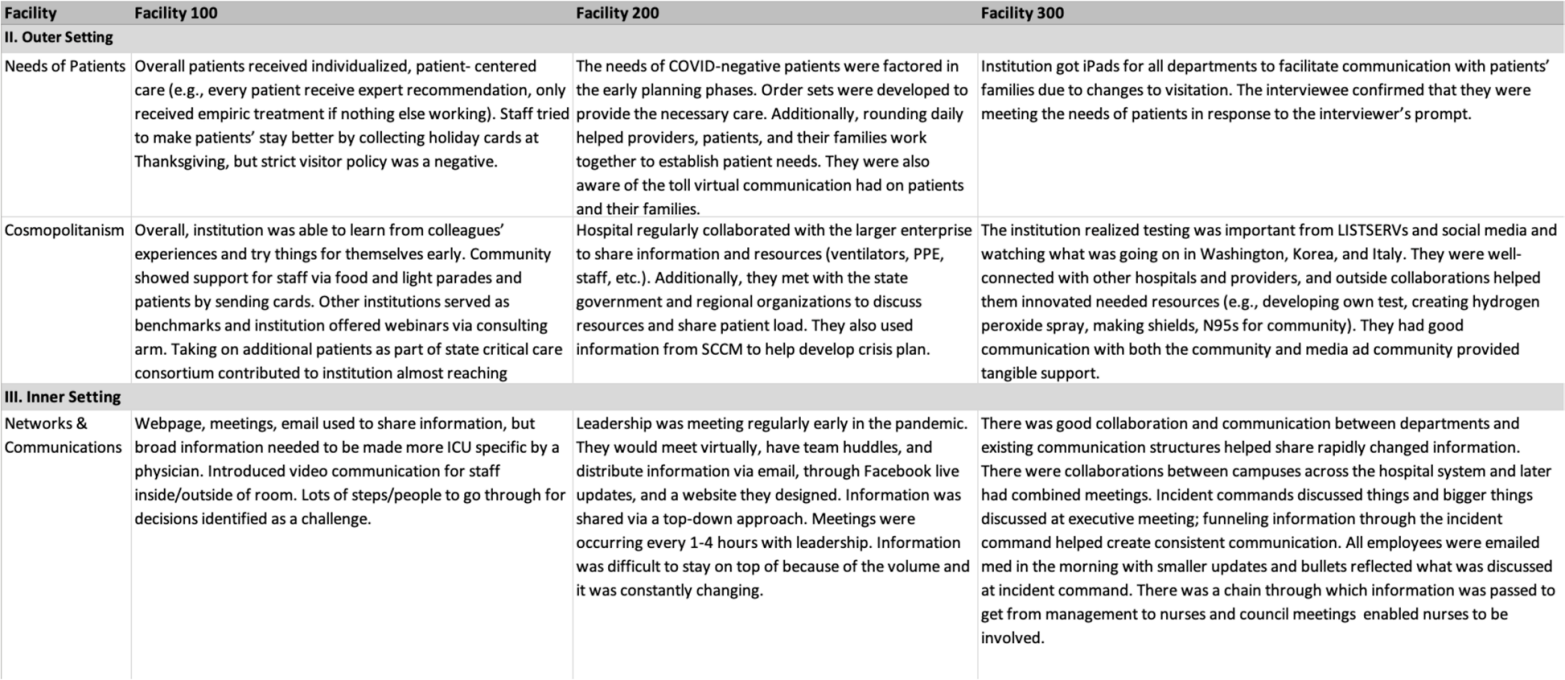
Analysis Matrix Table: used to compare construct summaries across facilities

**Fig. 4:**
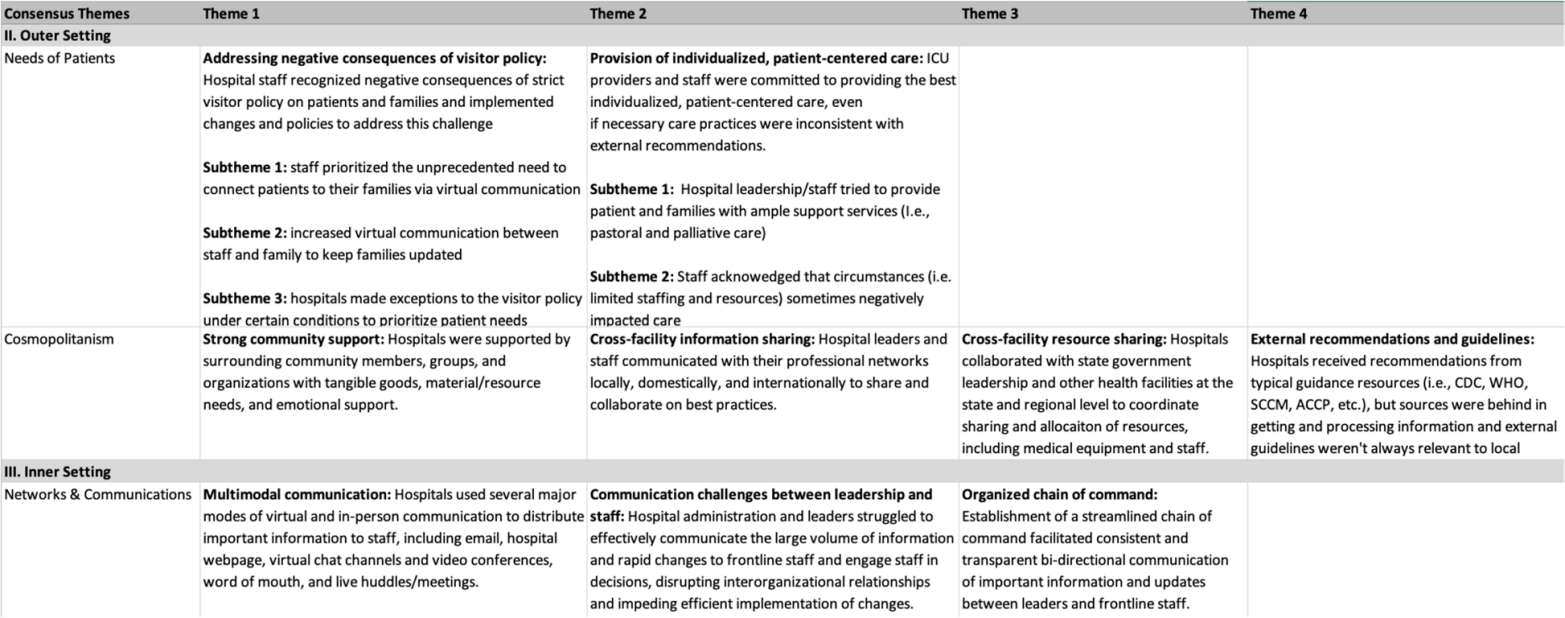
Consensus Theme Table: used to document, sort, and refine identified consensus themes for each construct

After coding was complete, the summary themes were aggregated by site to develop a memo report for each facility. Upon completion of facility memo reports, we extracted the information into an analysis matrix to compare summaries across sites (Fig. 3). For each CFIR construct, the analysts used the analysis matrix to independently review the construct summaries across sites and identify crosscutting themes. The analysts met to compare identified themes for the first four CFIR constructs and the qualitative lead constructed consensus themes for each construct based on these discussions (Fig. 4).

Due to the high agreement of themes generated across analysts, consensus meetings were not conducted to review the remaining constructs. Instead, the qualitative lead synthesized the themes independently identified by each analyst and developed consensus themes for each construct, which were reviewed, revised as necessary, and approved by the entire evaluation team. After sorting and consolidating the identified themes, this process resulted in 30 total themes, which were distilled into 12 strong themes that captured major identified implementation facilitators and barriers and mapped across the five CFIR domains and eleven CFIR constructs. We followed the consolidated criteria for reporting qualitative research (COREQ) checklist to develop the manuscript [17].

## Results

Of the 20 hospitals with the highest and lowest adjusted mortality quartiles recruited for this study, 17 facilities agreed to participate. The facilities were geographically distributed across the U.S. and varied in size, patient population, management, and funding structure (Table 2). Among these 17 sites, three contacted clinical leaders denied participation and 31 (15 ICU MDs and 16 ICU RNs) agreed to participate (Table 3). Thematic data saturation was achieved.

**Table 2:** (Results) Facility participant characteristics

Variable	Qualitative Sample (***N*** = 17^a^)
**Geographic region**	
**Northeast**	4
**Southeast**	7
**Midwest**	4
**West**	0
**Southwest**	2
**Financing source**	
**Government**	4
**Private individual**	2
**Not for profit**	13
**Other**	1
**ICU type**	
**Medical ICU**	8
**COVID-specific ICU**	6
**Mixed (medical-surgical)/Other**	2
**Total hospital beds**	
**≤ 250**	4
**251-500**	5
**501-750**	3
**> 750**	4
**Total ICU beds**	
**≤ 50**	6
**51-100**	5
**101-150**	1
**> 150**	4
**Total ICU units**	
**1-3**	6
**4-6**	6
**7-9**	1
**> 9**	3

^a^One site has missing demographic data because they did not respond to the 2020 Discovery VIRUS COVID-19 Registry survey

**Table 3:** (Results) Interview participant characteristics

Variable	Qualitative Sample (***N*** = 31)
**Age (years)**	
**20-35**	1
**36-50**	17
**51-65**	13
**Male**	13
**Female**	18
**Race/Ethnicity**	
**White/Caucasian**	24
**Asian (Asian American, Indian, Middle Eastern)**	6
**Hispanic/Latino**	2
**Professional degree**	
**MD/DO**	15
**RN (DNP, MSN, APRN, BSN, AND)**	18
**Other**	1

Of the 12 themes generated, six were related to implementation facilitators and six were related to implementation barriers. Facilitators promoted the implementation of new critical care practices while barriers hindered the implementation of new critical care practices. We organized these themes into the five CFIR domains: (1) Intervention Characteristics, (2) Outer Setting, (3) Inner Setting, (4) Characteristics of Individuals, and (5) Process. Within these domains, themes mapped to 11 distinct CFIR constructs (Table 4).

**Table 4:** (Results) Qualitative themes, organized by implementation facilitators/barriers across the CFIR domains

CFIR Domain	CFIR Construct	Implementation Determinant	Theme
**Intervention Characteristics**	Innovation Source	Facilitator	Practices informed by both external & internal sources
	Complexity	Barrier	Increased complexity of COVID-19 critical care practices
**Outer setting**	Cosmopolitanism	Facilitator	Strong partnerships with external stakeholders
	Cosmopolitanism	Barrier	Lack of consistent, reliable, & peer-reviewed guidance from trusted external resources
	Needs of Patients; External Policy	Barrier	Strict external infection prevention measures & policies that isolated patients
**Inner setting**	Networks & Communications	Facilitator	Coordinated & collaborative network/ communication structures
	Leadership Engagement	Facilitator	Strong leadership engagement, accessibility, & physical presence
	Available Resources	Barrier	Lack of PPE, medical equipment, ICU-trained staff, space, & technology
**Characteristics of individuals**	Knowledge & Beliefs about the Changes	Facilitator	Staff resilience & receptiveness to change
	Knowledge & Beliefs about the Changes	Barrier	Low staff morale & high burnout
**Process**	Engaging Staff	Facilitator	Effective staff engagement strategies
	Implementation Leaders	Barrier	Authoritarian decision-making

### Intervention characteristics

The first CFIR domain is related to the characteristics of the innovation being implemented. We identified two major characteristics of new critical care practices that evidently influenced their implementation: (1) the ‘Intervention Source’ of the care practice, or whether it was externally or internally developed; and (2) the perceived ‘Complexity’ of the care practice.

#### Facilitator: care practices that were informed by both external and internal sources were successfully adopted and adapted in local ICU settings

Especially given the unprecedented uncertainty around best critical care practices to treat COVID-19 patients, staff had increased confidence in implementing new practices that were externally endorsed and evidence-based. However, interviewees highlighted the need to critically evaluate external guidelines due to their rapidly changing and politically driven tendencies. Therefore, clinical leaders strived to select and implement practices that were informed by both external guidelines and internal review, testing, and refinement to ensure suitability to specific institutional context and needs.

*We were relying a lot on WHO and we were trying to follow CDC guidelines. But then, we had such great thought leaders from our infectious disease department and infection practices plus our laboratory practices. Either interpreting those guidelines or modifying to meet our needs as an institution. So, I think our ID and our IPAC lab folks, really pulled a lot of weight and were able to say, ‘Okay, this is what CDC says but are we gonna be more conservative than that, you know? This is their guidance, but how do we wanna interpret it and implement it. And two, is it the right implementation for our institution?’ I mean how do we adapt it, maybe is the better way to say it for our institutional needs. (ICU Supervisor, 101)*

#### Barrier: increased complexity of COVID-19 critical care practices hindered efficient implementation of procedures

Largely due to high viral infectivity and transmissibility of COVID-19 and consequent increased safety precautions for staff and patients, familiar practices such as intubation, proning, and physically transferring patients required more time and resources.

*When we intubate somebody, it was an ordeal. We had to have all of our equipment set up, all of our PPE on, and it was a whole process. It just took a lot longer and was a lot more complex than we normally did, a lot more resource intensive. (MD, 702)*

### Outer setting

The ‘Outer Setting’ domain includes relationships and interactions a hospital has within the larger economic, political, and social context in which it resides. Within this domain, we identified both implementation facilitators and barriers related to the CFIR construct ‘Cosmopolitanism’, which captures the degree to which an organization is networked with other external organizations. We also identified a barrier tied to ‘External Policies’ that influenced sites’ abilities to prioritize ‘Patient Needs’.

#### Facilitator: strong partnerships with external stakeholders, especially community leaders, local businesses, and other healthcare facilities, improved implementation efforts through promoting community support and cross-facility information and resource sharing

Participants revealed that established relationships with surrounding community members, groups, and organizations supported the implementation of new practices through providing needed resources and improving staff morale.

*There was really just outstanding communication with our community leaders and with our organization leadership. From the frontline worker perspective, it was just such an intense outpouring of support by our community whether it be people making headbands with buttons to hold up the masks or caps. The community rallied and brought just an incredible amount of drinks and food. It was unbelievable, just the outpouring. It was tangible as far as supplies, too. (ICU Director of Nursing, 301)*

Similarly, strong networks with government and other healthcare organizations supported implementation efforts through promoting the sharing of best practices and resources, including medical equipment and staff.

*We had really great support from our sister facilities. So, we didn’t purchase new things. But if I needed a food warmer, for whatever reason, I would just call another facility and they say, ‘Yep, we’ll have it on the loading dock. Sending someone over.’ They would borrow things out to me and that was really nice to know that they would do that. (COVID ICU Manager, 401).*

#### Barrier: strict external infection prevention measures and policies that isolated patients from providers and families hampered the delivery of patient-centered critical care

Guidelines around infection prevention and social-distancing from external governing agencies such as the Centers for Disease Control and Prevention (CDC) compelled hospital leaders to enforce strict measures that isolated COVID-19 patients away from both providers and family members. The switch to socially-distanced care was consistently cited as one of the most challenging critical care practice changes for all stakeholders to cope with. While the practice was critical to prevent COVID transmission, it hindered staff’s ability to address the holistic needs of patients and their loved ones. Specifically, isolation policies created communication challenges between patients, providers, and families that compromised the social-emotional aspect of patient-centered care.

*The physicians were not going into the rooms, so all the rounding was done outside the room. So, obviously that created some [patient-provider] relationship problems…we felt like the patients still deserve the same quality or standard of care that they had gotten prior to COVID and we felt like that was not happening. (RN, 901)*

In addition to the patient-provider communication barriers that resulted from these mandates, staff struggled with implementing effective methods for virtually communicating with families.

*The day-to-day flow with patients' families changed. That was very hard change for us being very patient and family-centered with our organization…With the change that occurred and families not being able to visit in the same way challenged us to really think outside the box with how we were going to proactively communicate. (ICU Director of Nursing, 301)*

#### Barrier: lack of consistent, reliable, and peer-reviewed guidance and information from trusted external resources impeded implementation of evidence-based practices

Participants explained the way in which uncertainty around best COVID-19 care practices was exacerbated by the unprecedented paucity of information from reputable sources.

*We found that our societies, like the American Medical Association, all the other typical societies, were behind in getting information and processing it, because they weren't on the frontlines. And all the frontline doctors didn't have time to do the studies or do the work to get this information to them. So we weren't getting guidance from our typical resources. (RN, 1101)*

In addition to hindering the implementation of evidence-based care for COVID-19 patients, the vacuum of information caused providers to experience frustration, fear, and distrust in decisions around best care practices, further impeding effective delivery of new care protocols.

*With the lack of information people started speculating, and if you start speculating then you begin to distrust the process...with the lack of information people were frustrated, and obviously scared because of the unknown. (MD, 402)*

### Inner setting

Within the third CFIR domain, which pertains to the structural characteristics, networks and communications, available resources, and culture within a hospital, we identified two facilitators and one barrier to implementation of new care practices. Major organizational factors that facilitated implementation efforts included strong ‘Networks and Communications’ and ‘Leadership Engagement’. The most significant barrier was lack of ‘Available Resources’.

#### Facilitator: coordinated and collaborative network and communication structures contributed to efficient integration of new care practices

In response to the pandemic, many facilities established an incident command center, which promoted consistent and transparent communication of important information between hospital leaders, managers, and frontline staff.

*I think having our incident command center there just to have information funneling through that structure was key to making sure we had good, consistent communication coming out. The cascade flow of information through the structure that we had in place was good. The collaboration, unbelievable collaboration –we already have great communication between our departments here but just the collaboration was at a new level. (ICU Medical Director, 302)*

Another common strategy used by clinical leaders to cultivate organized and collaborative communication structures was frequent multidisciplinary meetings and rounds, which facilitated productive discussion and implementation of new care practices.

*The key to our not success, but our survival was more the communications that were set up between us using the multidisciplinary rounds, so that we were able to share information throughout our hospital system. We were able to pass it along to everyone, so we could use a larger brain to troubleshoot ideas or bounce ideas or figure things out, as an ICU, we weren't left standing by ourselves trying to figure this out. (MD, 1102)*

#### Facilitator: strong leadership engagement, accessibility, and physical presence facilitated implementation efforts through supporting the needs of patients and frontline staff

Interviews revealed that the daily presence and direct assistance of mid- and high-level leaders on the floor was instrumental in cultivating a culture of teamwork and addressing implementation challenges related to the increased complexities of procedures and constrained resources.

*It was very much a leadership from the front style, literally elbow to elbow with the staff in proning these patients, in intubating these patients and transporting these patients even. There was no hierarchical feel to it, because all of us were learning. (MD, 1702).*

The immense appreciation frontline staff had for leadership presence is a testament to the meaningful impact it had on the daily delivery of care through reducing the overwhelming burdens felt by ICU frontline staff.

*To see [hospital leaders] walking through and just the recognition rounds and just the appreciation rounds, that was, again, tangible because when you're in that environment, it was very overwhelming for our teams and just to be able to have them there and round through, that was very much appreciated. (RN, 300)*

#### Barrier: lack of adequate resources, including PPE, medical equipment, ICU-trained staff, space, and technology, often hindered implementation of care

Participants described the ways in which unprecedented resource challenges, especially PPE and medical equipment, disrupted implementation of care practices and infection prevention measures.

*We’re reusing PPE. We never did anything like that before…I’ve been in the ICU for 30 years and never ever was in a situation where I had to reuse PPE... We had to change how often we were changing IV tubing because there was a national shortage of IV tubing, so a lot of our infection prevention measures changed because of reusing the PPE. (ICU Director of Nursing, 301)*

In addition to inadequate supplies of necessary protective and medical equipment, major staffing shortages during the pandemic impacted delivery of care.

*By Memorial Day we had every bed full and I didn’t have half the amount of nurses I needed... So, now I have a huge nursing shortage on top of a huge nursing shortage. (COVID ICU Manager, 401).*

### Characteristics of individuals

Through elucidating the specific beliefs and characteristics of individuals involved with implementing the intervention or practice, the fourth CFIR domain recognizes that people are not passive recipients of innovations, but rather intimately engage with and influence implementation efforts. The vital role of the ‘Knowledge and Beliefs’ of frontline staff in the implementation of new care practices was evident throughout interviews.

#### Facilitator: staff resilience and receptiveness to change promoted successful implementation of new care practices despite barriers

Participants highlighted the way in which staff’s passion for and dedication to critical care supported implementation efforts through fostering a sense of ownership in caring for patients and the overall community.

*Besides the fear and the grief, and some of the emotional toll, there was also a call to arms. Like I was invigorated... I don't know, my love for critical care was reaffirmed again. (MD, 1102)*

This ownership and commitment also reduced barriers associated with staffing shortages.

*The easiest thing was finding people to cover. The vast majority of my colleagues and coworkers, would say, ‘Yeah, this is our job’ and they stepped up to the plate willingly. I think there was almost a sense of pride in that. (MD, 402)*

#### Barrier: Low staff morale and burnout, largely caused by fear and anxiety, staffing shortages, change fatigue, and emotional and physical exhaustion, hindered delivery of quality critical care

ICU staff reported experiencing anxiety due to the uncertainty of the pandemic and fear of exposing themselves and their families to the virus.

*It was the fear of the unknown. What would the patients be like? Would we have the tools and resources to care for these patients and the anxiety around the unknown of whether or not we would be able to care properly for the patients and then our own safety, both our physical and psychological safety. (RN, 601)*

These negative emotions, exacerbated by feeling overworked, led to remarkable staff burnout, which participants described as disturbing both the healthcare system as a whole and uptake of local ICU practices.

*It wasn't until I felt myself break and say I have to step away, did I realize how bad the burnout was. The staff came and really broke down to me...The burnout is real, the burnout, the PTSD, it's real, and the toll it took in healthcare is real. (COVID ICU Manager, 401).*

### Process

The last CFIR domain includes factors involved in the active change processes aimed at achieving individual and organizational implementation of practices. Our data revealed that productively ‘Engaging Staff’ in the change process facilitated implementation efforts, while siloed decision-making by ‘Implementation Leaders’ hindered implementation efforts.

#### Facilitator: effectively engaging frontline staff throughout the implementation process fostered a robust, productive, and collaborative workforce highly capable of implementing new practices

Clinical leaders who effectively engaged staff in change processes often did so through soliciting and responding to staff needs and acknowledging staff efforts. Leadership sought out staff input during huddles, rounds, and town hall meetings and incorporated their feedback into decisions about practice changes. Engaging staff in decisions not only garnered staff buy-in around new practices but also generated valuable insights that helped guide selection and integration of practices.

*Someone from the leadership team would huddle directly with the staff so that there could be bi-directional communication, questions and answers, if you will, because there were always lots of insightful questions. That's how we gained a lot of our insight was at those huddles. (RN, 601)*

Even when leaders couldn’t fully address staff needs, they maintained engagement and support through validating staff concerns and appreciating their efforts.

*Connecting with staff was important. Validating their concerns was very important. Because we didn’t have solutions to every problem that people raised, being available was such a key thing. Then doing those little things, like ensuring that we acknowledged people when they did a great job taking care of a patient. (MD, 202).*

#### Barrier: governing the change process by authoritarian decision-making and lack of collaboration across leadership levels and departments hindered implementation efforts

Participants described the challenges associated with this process barrier, expressing the frustrations provoked when hospital leaders and administrators made unilateral decisions about practice changes without consulting providers and frontline staff.

*Some frustration, of course, that’s expected when you are not involved with the decisions or know why were they made…We as critical care physicians were never involved much to be honest, like when they made the decision, are we going to open this unit, are we going to open, it was mainly infectious control and the CMO. I don’t think us as critical care physician had any role in that. (MD, 1502)*

This authoritarian decision-making process governed by removed high-level hospital leaders also produced problematic decisions about practice changes that were not based on the realities of clinical practice on the floor, thereby exacerbating implementation challenges and tensions between administrators and frontline staff.

*The Administrators think oh these things are the best idea ever. But yet they don't come to the bedside and they have no idea about the reality of it…The administrator, I knew how they would think. And I'm like you know what? I'm not even talking to them. Absolutely not. We are clinicians, we're going to make this decision. (ICU Director, 1202)*

## Discussion

This study sought to identify and evaluate implementation factors, including facilitators and barriers, that influenced the development and uptake of new ICU care practices in response to COVID-19. We generated six themes under implementation facilitators and six themes under implementation barriers. Within the CFIR domain of ‘Intervention Characteristics’, we identified a facilitator linked to the CFIR construct ‘Innovation Source’, which captures the way in which utilization of both external and internal guidelines supported sites in the challenge to select and implement best critical care practices in a time of unprecedented uncertainty. A strong barrier was related to the construct of ‘Complexity’, representing how the increased complexity of patient care due to the high volume and acuity of COVID-19 patients hindered efficient implementation of familiar care procedures such as intubation and proning. These findings, illustrating how aspects of the care innovation itself affect implementation effectiveness, are consistent with other studies that have explored how innovation characteristics influence implementation [18–20].

Similarly, this study corroborated evidence that ‘Outer Setting’ characteristics, including overall networks with external stakeholders, ‘Needs of Patients’, and ‘External Policy’, have important implications on implementation effectiveness [18, 21, 22]. While our findings suggest that strong networks with external entities facilitated implementation of new care practices through cross-facility resource and information sharing, they also reveal the challenges associated with reliance on outside sources for guidance. For example, interviewees highlight how external information was often contradictory, unreliable, and lacked peer-review, hampering the delivery of evidence-based and patient-centered critical care. This exemplifies the need for stronger internal research and innovation efforts as well as more coordinated processes for vetting and disseminating COVID-19 data and information across public health agencies, national and local governments, hospital associations, and nonprofit medical organizations.

Within the ‘Inner Setting’, we found that strong ‘Network and Communications’ structures that utilized multimodal communication strategies, frequent multidisciplinary meetings, and an organized chain of command, facilitated implementation effectiveness. Ongoing staff communication and solicitation of feedback have also been cited by other studies to improve implementation success [23–25]. Supporting the findings of previous studies, the crucial role of strong communication and feedback between leadership and staff highlights the benefit of developing organized communication and feedback structures [18]. Establishing mechanisms that support two-way communication also enable clinical leaders to engage frontline staff by soliciting their input and responding to needs and concerns. Engaging staff through incorporating their feedback into decision-making processes was identified as an implementation facilitator within the ‘Process’ domain. This has also been cited by previous literature as an effective strategy to promote employees’ readiness towards change through making them feel needed, essential, and valued [26].

The major barrier within the ‘Inner Setting’ domain – lack of ‘Available Resources’ – highlights the universal need for greater dedication of healthcare resources to ICU settings and novel strategies to address resource constraints, such as the establishment of supply chain protocols and standardized material handling procedures that monitor hospital resource needs. An implementation facilitator that counteracted resource constraint barriers was ICU staff resilience and receptiveness to change, which corresponded with the ‘Characteristics of Individuals’ CFIR domain. Staff dedication and ownership to provide quality patient care despite immense challenges was instrumental in filling staffing gaps, alleviating staff anxiety and burnout, and maintaining a positive work atmosphere, all of which promoted successful implementation of new ICU practices. Importantly, this staff commitment was cultivated by establishment of teamwork and camaraderie, which previous studies have similarly identified as facilitators of the implementation process [27–30].

This suggests that to address barriers related to low staff morale and burnout and promote implementation effectiveness, ICU clinical leaders should strive to cultivate a strong culture of interprofessional teamwork. Some evidence-based strategies to support leaders in this mission include establishment of common performance goals, clear communication of a shared vision [31], employing a system thinking approach and encouraging communities of practice [32], and using interprofessional team-based training [33]. Through discouraging a silo working culture, these strategies simultaneously address problematic authoritarian decision-making and lack of collaboration, which were identified as implementation barriers within the ‘Process’ domain.

### Strengths and limitations

Using the CFIR to guide our analysis was instrumental in rapidly identifying and organizing themes into multi-level intervention factors that evidence shows are key influences on implementation effectiveness [2]. Therefore, our study demonstrates the value of using the CFIR to systematically evaluate facilitators and barriers to implementing changes in healthcare practices across diverse, multi-level contexts. Furthermore, the use of multiple analysts to refine the codebook and establish coding consensus before single-coding analysis helped to minimize measurement error through ensuring consistency of categorization using the CFIR codes and increased the reliability of our findings through checking interpretations against the data from a variety of perspectives. Moreover, discussing coding disagreements through our consensual qualitative approach enabled us to identify codes that were not sufficiently well-defined or relevant to the data, thereby producing greater conceptual clarity and rigorous analysis.

A major limitation of this study was that the CFIR was applied retroactively for data analysis but was not formally used to guide data collection. It would have been beneficial to use the CFIR to inform development of the interview guide to identify salient framework constructs to probe for during interviews and further strengthen the theoretical foundation and continuity across data collection, analysis, and interpretation. Although previous studies have found that using the CFIR to guide data collection has advantages over applying the CFIR in data analysis only [34], we were still able to achieve a comprehensive alignment between the interview guide questions and CFIR constructs, enabling a conceptually coherent, systematic, and rigorous exploration of the data. Another limitation was the inherently more deductive and explanatory nature of rapid template analysis, compared to the inductive and exploratory qualities of traditional qualitative methods. Application of the CFIR to develop a priori codes might have obscured qualitative themes that were relevant to our research question but not explicitly applicable to the CFIR domains and constructs. To address this limitation while balancing the need to deliver timely findings, we used a deductive template to structure analysis but maintained an iterative coding and conceptualization process that enabled the generation of themes that emerged more inductively from the data.

Lastly, there were a few limitations with the recruitment and sampling strategies that influenced the generalizability of our conclusions. For example, recruiting participants on the bases of Discovery VIRUS COVID-19 Registry involvement and clinical leadership allowed us to capture the valuable perspectives of clinical leaders, but the results may not accurately reflect the important and nuanced experiences of frontline workers across the country. Similarly, although both medical and nursing ICU directors were invited to participate at each facility, some facilities only had representation from one participant, thereby limiting the diversity of perspectives and richness in the data. Finally, selecting hospitals from only the lowest and highest adjusted mortality quartiles may exclude themes that are distinct to hospitals with average mortality rates.

## Conclusions

The COVID-19 pandemic has resulted in instrumental changes to society and the practice of critical care medicine. Based on our findings, we can draw a few important conclusions to address our study objective of exploring effective ICU clinical practices and change management strategies during COVID-19. First, major external barriers to implementation efforts included lack of access to information and material resources and strict guidelines that were difficult to adapt to local contexts. Major internal barriers included various organizational factors related to leadership and communication styles, particularly poor leadership engagement, lack of collaboration across diverse stakeholders, and fragmented network and communication structures. Despite these obstacles, resilient ICU leaders and staff developed novel solutions to deliver best possible care to COVID-19 patients. There were several organizational factors that supported staff in this challenge, including: (1) Robust networking and communication with diverse internal and external stakeholders; (2) Collaborative and streamlined processes for deciding and implementing changes; and (3) Engaged leaders who fostered a culture of teamwork and were committed to meeting the holistic needs of frontline staff and patients.

The analysis presented here is only the first stage of this research project. Next, we will sort the participating facilities by their COVID-19 mortality rates so that we can identify patterns of distinguishing themes between hospitals with low versus high mortality outcomes. This differential analysis will allow us to explore how variability in ICU practices, organizational factors, and implementation strategies relate to mortality rates. Ultimately, we aim to harness our findings to support clinical leadership with valuable insight on recommended practices and change management strategies.

## Supplementary Information

**Additional file 1.** Semi-structured interview guide (PDF).

**Additional file 2.** Consolidated criteria for reporting qualitative research (COREQ) checklist.

## Data Availability

The datasets used and/or analyzed during the current study are not publicly available due to privacy and confidentiality of our research participants, but are available from the corresponding author on reasonable request.

## Abbreviations

COVID-19: Coronavirus disease 2019
ICU: Intensive care unit
CFIR: Consolidated Framework for Implementation Research
SCCM: Society of Critical Care Medicine
Discovery VIRUS COVID-19 Registry: Discovery Viral Infection and Respiratory Illness Universal Study COVID-19 Registry
CDC: Centers for Disease Control and Prevention
