# Supplementary material for: Facilitators and barriers to the implementation of new critical care practices during COVID-19: a multicenter qualitative study using the Consolidated Framework for Implementation Research (CFIR)
Source: BMC Health Serv Res. 2023 Mar 20;23:272. doi: 10.1186/s12913-023-09209-w (PMC10026230; doi:10.1186/s12913-023-09209-w)
Supplement: Supplementary file 1 — Additional file 1. Semi-structured interview guide (PDF). [file 12913_2023_9209_MOESM1_ESM.pdf]

Facilitators and Barriers to the Implementation of New Critical Care Practices during COVID-19: A Multicenter Qualitative Study using the Consolidated Framework for Implementation Research (CFIR)

Interview Guide

Screening questions:

1. Do you feel comfortable conducting the entire interview in English?
2. Are you over the age of 18?
3. Are you currently in an ICU leadership role (e.g., Medical Director, Nursing Director, Administrator)
4. Have you been working in the ICU since January 2019?

(Note: If any of the above answers are "no," participant does not meet eligibility criteria and cannot enroll in the study.)

Demographics

1. What is your age?
2. What is your gender?
3. What is your race?
4. How long have you been in your position?
5. What is your professional degree(s)?

ICU-specific questions

1. How did COVID effect your usual ICU practices?
    a. Probes:
        i. What were some of the biggest changes? (e.g., sites of care, team structure, rounding habits, use of non-invasive respiratory support, use of mechanical ventilation, use of sedatives, use of adjunctive treatments (such as prone position, inhaled nitric oxide, paralytics), use of tracheostomy, involvement of adjunctive care teams (PT/OT) and consultants, family involvement, end of life care)
        ii. What practices stayed the same?
2. When changing specific practices, how did you make these decisions?
    a. Probes:
        i. Who was involved in making these decisions?
        ii. Why were they involved?
3. What was the most challenging part of making these decisions?
4. How were these changes communicated to staff?
5. How did the staff respond to these changes?
6. Which practice changes were easiest to implement?
    a. Probes:

        i. What made these changes easy to implement? (e.g., leadership support, teamwork, staff morale, resources)
        ii. What resources were provided by your institution? By the external community?
7. Which practices changes were hardest to implement?
    a. Probes:
        i. What made these changes difficult to implement? (e.g., leadership support, teamwork, staff morale, resources)
        ii. What types of support would have made it easier?
8. In thinking about all the changes you've had to make, what strategies, tools and/or resources have been the most helpful?
9. <u>Institution-specific questions</u>
10. How did institutional regulations or external guidelines effect the way you provided care to COVID patients?
    a. Probes:
        i. What was helpful?
        ii. What was challenging?
11. How has your institution communicated and shared information regarding the pandemic?
    a. Probes:
        i. What has worked well?
        ii. What hasn't worked well?
12. In what ways was your institution prepared to deal with the COVID pandemic? Least prepared?
13. What could your institution do to be more responsive to the needs of COVID patients?
